# Determining highly pathogenic H5 avian influenza clade 2.3.2.1c seroprevalence in ducks, Purbalingga, Central Java, Indonesia

**DOI:** 10.14202/vetworld.2020.1138-1144

**Published:** 2020-06-18

**Authors:** Imas Yuyun, Hendra Wibawa, Gunawan Setiaji, Tri Anggraeni Kusumastuti, Widagdo Sri Nugroho

**Affiliations:** 1 Magister Sain Veteriner, Faculty of Veterinary Medicine, Universitas Gadjah Mada, Yogyakarta, Indonesia; 2 Directorate of Animal Health, Directorate General of Livestock and Animal Health Services, Jakarta, Indonesia; 3 Disease Investigation Center, Wates, Yogyakarta, Indonesia; 4 Directorate of Veterinary Public Health, Directorate General of Livestock and Animal Health Services, Jakarta, Indonesia; 5 Department of Socio Economic, Faculty of Animal Science, Universitas Gadjah Mada, Yogyakarta, Indonesia; 6 Departement of Veterinary Public Health, Faculty of Veterinary Medicine, Universitas Gadjah Mada, Yogyakarta, Indonesia

**Keywords:** avian influenza, ducks, farming system, highly pathogenic avian influenza H5, risk factors, seroprevalence

## Abstract

**Background and Aim:**

In Indonesia, highly pathogenic avian influenza (HPAI) H5N1 outbreaks in poultry are still reported. The disease causes a decrease in egg production and an increase in mortality; this has an impact on the economic losses of farmers. Several studies have considered that ducks play a role in the HPAI endemicity in the country; however, little is known about whether or not the type of duck farming is associated with HPAI H5 virus infection, particularly within clade 2.3.2.1c, which has been predominantly found in poultry since 2014. A cross-sectional study was conducted to determine the HPAI seroprevalence for H5 subtype clade 2.3.2.1c in laying ducks that are kept intensively and nomadically and to determine the associated risk factors.

**Materials and Methods:**

Forty-nine duck farmers were randomly selected from ten sub-districts in Purbalingga District, Central Java, Indonesia; a cross-sectional study was implemented to collect field data. Based on an expected HPAI prevalence level of 10%, estimated accuracy of ± 5%, and 95% confidence interval (CI), the total sample size was calculated at 36 individuals. Samples must be multiplied by 7 to reduce bias; thus, 252 ducks were taken as samples in this study. Considering that the maintenance and duck handling were uniform and farmers complained that the effect of activity to take duck samples would reduce egg production, this study only took samples from 245 ducks (oropharyngeal swabs and serum). Those samples were taken from five birds on each farm. Hemagglutination inhibition tests examined the serum samples for HPAI H5 Clade 2.3.2.1c, and pool swab samples (five swabs in one viral media transport) were examined by real-time reverse transcription-polymerase chain reaction (qRT-PCR) test for influenza Type A and H5 subtype virus. Information regarding farm management was obtained using a questionnaire; face-to-face interviews were conducted with the duck farmers using native Javanese language.

**Results:**

Serum and swabs from 245 ducks were collected in total. For individual birds, 54.69% (134/245) of serum samples were H5 seropositive. Seroprevalence among nomadic ducks was 59.28% (95% CI: 0.48-0.61), which was higher than among intensively farmed ducks (48.57%, 95% CI: 0.38-0.58). Farm-level seroprevalence was 50% (95% CI: 0.30-0.69) for nomadic ducks but only 28.57% (95% CI: 0.11-0.51) for intensively farmed ducks. The farm-level virus prevalence (proportion of flocks with at least one bird positive for influenza Type A) was 17.85% (95% CI: 0.07-0.35) for nomadic ducks and 4.76% (1/21) for intensively farmed ducks (95% CI: 0.008-0.23). All influenza Type A positive samples were negative for the H5 subtype, indicating that another HA subtype AI viruses might have been circulating in ducks in the study area. A relationship between duck farms that were H5 seropositive and their maintenance system was present; however, this relationship was not significant, the nomadic duck system detected 2 times higher H5-seropositive ducks than the intensive farming system (OR: 2.16, 95% CI: 0.33-14.31).

**Conclusion:**

This study found that the seroprevalence of HPAI in the duck population level in Purbalingga was 54.69% and demonstrated that the nomadic duck farming system was more likely to acquire HPAI H5 infection than the intensive farming duck system. Other risk factors should be further investigated as the diversity of the farming system is partially related to HPAI H5 infection.

## Introduction

Avian influenza (AI) is a viral disease caused by the influenza Type A virus, which infects the respiratory, digestive, reproductive, and nervous systems of various avian species. AI viruses vary significantly in their ability to cause disease (pathogenicity) and their ability to spread among birds. Wild bird species usually do not show clinical symptoms of the disease; however, some AI virus strains can cause severe illness and death in chickens, ducks, and turkeys [1]. Based on the pathogenicity in chickens, the AI virus can be divided into two types: Highly pathogenic AI (HPAI) and low pathogenic AI (LPAI) [1,2]. Although there have been many combinations of AI subtypes (H1-H18 and N1-N11), HPAI outbreaks in poultry are mainly caused by H5 or H7 subtype viruses [3,4]. HPAI outbreaks in poultry in Indonesia were first reported in 2003 [1,5]. The majority of disease outbreaks in poultry were reported from gallinaceous species (chickens, quails, and turkeys) from different production sectors due to infection through the H5N1 subtype virus of clade 2.1.3. However, since the incursion of the H5N1 clade 2.3.2.1c virus in 2014, the poultry outbreaks were not only found from gallinaceous birds but also reported from waterfowl, including ducks, Muscovy ducks, and other aquatic birds [6]. In domestic ducks, the H5N1 clade 2.3.2.1c caused high mortality in young ducks and a drop in egg production in laying ducks [7].

Transmission of AI infection occurs through direct contact from infected birds through respiratory, conjunctival, or nasal mucus and feces; or indirectly through dust, feed, drinking water, farm workers, equipment, pens, shoes, clothes, and vehicles contaminated with AI virus and infected live chickens [1,3,8]. Waterfowl, including ducks and Muscovy ducks, can act as carriers of the virus without showing clinical symptoms; waterfowl usually act as a source of transmission and infection to chicken and turkey farms. It is unknown whether vertical or congenital transmission occurs because there is no scientific or empirical evidence [1,9,10]. The H5 subtype virus of clade 2.3.2.1c has mainly been found in poultry in Indonesia since 2014; there have not been many studies investigating whether ducks play a role in the endemicity of HPAI in Indonesia. Purbalingga was selected as a representative district for endemicity based on the final report of the HPAI endemicity study in Indonesia [11], conducted by the Directorate General of Livestock and Animal Health Services, Ministry of Agriculture, in collaboration with Food Agriculture Organization ECTAD Indonesia between 2016 and 2017. HPAI cases have been reported in this district each month throughout consecutive years; the district has a poultry population representative of both commercial and backyard poultry keeping, with a mixture of chicken and duck farms, collector yards, and live bird markets trading in poultry. The study results confirmed that the area, as a representative of Java, is still endemic for HPAI. The study also stated that the virus was detected in all enterprise types, including commercial farms, live bird markets, collector yards, nomadic duck flocks, and villages. The HPAI viruses were confirmed in layer, broiler, duck, and kampong chickens [11].

A previous study showed that scavenging ducks (ducks kept to wander in the paddy field or nomadic system ducks) are a potential source of HPAI infection for backyard chickens [10], but specific control measures for HPAI cases in nomadic duck farms have not been developed. The following encompasses a cross-sectional study to determine the H5 clade 2.3.2.1c seroprevalence in laying ducks in the Purbalingga District of Central Java, Indonesia, also to investigate whether the farming system (intensive and nomadic) has an association with HPAI H5 subtype clade 2.3.2.1c infection on duck farms. Insight into HPAI control in ducks can be gained from this study. Previous studies have focused only on the epidemiology of the older virus clade 2.1.3 [10,12]; however, these outbreaks have rarely been detected in poultry in Indonesia.

This study was conducted to determine the HPAI seroprevalence for H5 subtype clade 2.3.2.1c in laying ducks that are kept intensively and nomadically and to determine the associated risk factors.

## Materials and Methods

### Ethical approval and informed consents:

All procedures performed in this study, including the collection of serum and oropharyngeal and cloacal swabs samples were in accordance with Animal Care and Use Committee, Faculty of Veterinary Medicine, Universitas Gadjah Mada Indonesia (Approval no. 0028/EC-FKH/Int/2019).

All human subjects gave their consent for the collection of information, with the agreement that any identifying details of the individuals would not be published.

### Study area

The present study was undertaken in March 2019 as a recommendation research of the HPAI endemicity study of the previous research conducted in 2016-2017 in Purbalingga, by the Directorate of Animal Health, Ministry of Agriculture, Republic of Indonesia, and in collaboration with the Food Agriculture Organization. Purbalingga District, in Central Java Province, was selected for the study area given that the district is quite representative of poultry market chains, including native backyard chickens, commercial farms, nomadic ducks, poultry collectors, and live birds markets.

**Figure-1:**
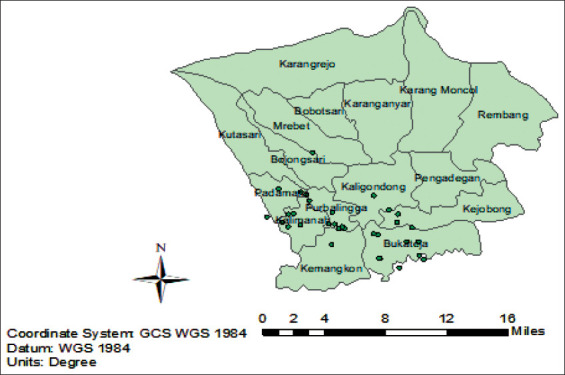
Duck farms sample location in Purbalingga District, Central Java, Indonesia.

Field sampling was conducted on small and medium scale commercial sectors (sector 3), which are maintained intensively and nomadically in ten sub-districts in Purbalingga: Kaligondang, Padamara, Purbalingga, Kemangkon, Mrebet, Kejobong, Kalimanah, Rembang, Bobotsari and Bukateja, Central Java, Indonesia (Figure-1).

### Study design and study population

This study was conducted on 21 intensive farming duck farms and 28 nomadic duck farms (based on data from Agricultural Services of Purbalingga District in 2016). The average scale of the animal farming business was 100-500 heads/farm, and the duration of maintenance ranged from 14 to 20 months with egg production between 60% and 70%.

Based on the expected HPAI prevalence of 10%, an estimated accuracy of ± 5%, and 95% confidence interval (CI), the total sample size was calculated to be 36, and according to a previous study [13], samples were to be multiplied by 7 to reduce bias; thus, 252 ducks were taken as samples in this study. Considering that the maintenance and duck handling were uniform and farmers complained that the effect of activity when taking samples would reduce egg production, this study only took 245 samples (oropharyngeal swabs and serum) from 49 duck farmers distributed around ten sub-districts. The samples were taken from five birds on each farm.

The selection of farms was carried out by random sampling. A sampling of ducks at the farm level was carried out by visual judgment, i.e., looking at ducks to find those that had symptoms of AI illness; all samples were collected in March 2019. Management information was obtained by a face-to-face questionnaire that was administered to the duck farmers in their native Javanese language.

### Laboratory testing

Samples were collected by Veterinary staff officers of the Agricultural Services of Purbalingga District and sent to the Disease Investigation Center, Wates, Yogyakarta Province. Serum samples were tested for antibodies to AI H5-subtype using the hemagglutination inhibition (HI) test. H5 clade 2.3.2.1c antigen for HI tests was used because the majority of circulating H5 virus in Indonesia is clade 2.3.2.1c, which is the only H5 virus clade detected in Central Java since 2015. Swabs were tested as pool samples (from five birds) usingreal-time reverse transcription-polymerase chain reaction (qRT-PCR) technique for the detection of influenza Type A and H5 subtype viruses.

All samples were obtained from HPAI-unvaccinated birds, any farms with at least one bird that showed as HI-H5 seropositive (HI titer ≥16 [2]) was considered an HPAI-positive (infected) farm. HPAI-negative farms had no HI-H5 seropositive results during the study. Based on farm serology diagnostic result, we performed qRT-PCR diagnosis. The qRT-PCR results were not included in the analysis because all the samples were negative for the H5 subtype virus even though some samples influenza Type A positive.

### Statistical analysis

Data from laboratory test results and questionnaires were combined in Excel 2013 for descriptive analysis. Data were collected and analyzed using Epi Info™ 7 (CDC) (https://www.cdc.gov/epiinfo/support/downloads.html) for binomial logistic regression analysis to determine the association of risk factors to HPAI H5N1 infection (seropositivity).

## Results

### Descriptive analysis

Farmers of laying ducks in Purbalingga District were from various backgrounds. In terms of education, 57.14% (28/49) of farmers only had an education up to elementary school level, 20.40% (10/49) continued to junior high school, 14.28% (7/49) gained a high school education, and 4.08% (2/49) were educated to the undergraduate level. Of the 49 duck farmers, only one farmer had never been to school formally. Most nomadic duck farmers only receive an elementary school education, 38.77% (19/49), while 20.40% (10/49) of intensive duck farmers had a better formal education, including up to undergraduate level.

The average farmers had more than 5 years of experience raising ducks (69.38%), while 8.16% and 22.44% of farmers had an experience of between 4 and 5 years and <3 years, respectively.

The average number of ducks raised by farmers was 200-500 ducks (21/49 or 42.85%). A total of 28.57% (14/49) maintained <100 head of ducks. Farmers who maintained between 100 and 200 animals were 22.44% (11/49), and only 6.2% (3/49) of duck farmers kept more than 500 ducks. A total of 36.73% (18/49) of farmer-maintained ducks were aged between 11 and 18 months, 34.69% (17/49) maintained ducks older than 18 months, and 28.57% (14/49) maintained young ducks, aged between 6 and 10 months. Most of the duck farmers showed that their egg production reached 70%; they did not conduct vaccination for disease prevention, particularly for AI.

The treatment of sick ducks was different in nomadic farmers, 46.42% (13/28) showed that sick ducks were treated with herbal medicine without a proper dosage, 32.14% (9/28) sold the sick ducks, 14.28% (4/28) of farmers left the ducks to die, and the farmers’ family consumed the sick duck 7.14% (2/28). Intensive duck farmers sold or treated their sick ducks, as many as 47.61% (10/21) of farmers sold their sick ducks, and the others claimed to self-treat with herbal medicines with unclear doses.

Disposal of duck carcasses also differed between nomadic and intensive farmers; 52% (11/21) of intensive duck farmers buried their dead ducks, while 28% (6/21) were burned, and 19% (4/21) of farmers dumped the carcasses into the nearest river. The number of nomadic farmers who disposed of duck carcasses using the river was greater than that of intensive farmers, being 53.57% (15/28). Only 32.14% (9/28) of nomadic farmers buried their carcasses, and 14.28% (4/28) burned ducks near their homes.

### HPAI (H5) seroprevalence and PCR

Table-1 shows serum samples from ten sub-districts in Purbalingga that had seropositive results for the HI test against the HPAI H5 antigen. A titer ≥16 of HI was considered positive [2]. The percentage of seropositive samples from each sub-district: Bukateja (3.27%), Purbalingga (2.45%), Kemangkon (2.45%), Rembang (2.45%), Kaligondang (2.04%), Kejobong (2.04%), Padamara (1.22%), Kalimanah (1.22%), Bobotsari (0.41%), and Mrebet (0.41%). The farm-level seroprevalence was 17.96% in total from ten sub-districts in Purbalingga. Table-2 shows bird- and farm-level H5 clade 2.3.2.1c seroprevalence in the study area. Based on maintenance systems, the farm-level seroprevalence of nomadic duck farms was higher (50%, 14/28) than that of intensive duck farms (28.50%, 6/21). The bird-level seroprevalence was 59.28% (83/140) for the nomadic system and 48.57% (51/105) for the intensive system.

**Table-1:** Number of serum and swab samples per district and test results (hemagglutination-inhibition and polymerase chain reaction) for avian influenza.

Sub-district	Number of serum samples tested	Hemagglutination-inhibition seropositive (%)		Number of swab samples tested	Polymerase chain reaction positive for influenza A(%)	
	
		Nomadic	Intensive		Nomadic	Intensive
Bukateja	50	1.63	1.63	10	2.04	2.04
Kaligondang	30	0.82	1.22	6	2.04	0.00
Kalimanah	15	1.22	0.00	3	0.00	0.00
Padamara	15	0.41	0.82	3	2.04	0.00
Purbalingga	35	1.22	1.22	7	2.04	0.00
Kejobong	30	0.41	1.63	6	0.00	0.00
Kemangkon	30	2.04	0.41	6	2.04	0.00
Rembang	30	2.45	0.00	6	0.00	0.00
Mrebet	5	0.00	0.41	1	0.00	0.00
Bobotsari	5	0.41	0.00	1	0.00	0.00
Total	245	10.61	7.35	49	10.20	2.04

**Table-2:** Bird- and farm-level H5 clade 2.3.2.1 seroprevalence in the study area.

Duck maintenance system	Numbers of farms samples	Numbers of farms showed seropositive (%)	Number of ducks	Numbers of duck showed seropositive (%)
Nomadic	28	14/28 (50.00)	140	83/140 (59.28)
Intensive	21	6/21 (28.50)	105	51/105 (48.57)

Further analysis of molecular testing showed that the percentage of positive qRT-PCR samples for Influenza A from each sub-district was: Bukateja (4.08%), Kaligondang (2.04%), Padamara (2.04%), Purbalingga (2.04%), and Kemangkon (2.04%), the other sub-district showed a negative result. The samples with cycle threshold (Ct) value <40 were considered positive for the AI virus, and this was adhered to for HPAI subtype detection using H5 specific primer and probes [3,14,15].

### Risk factor

Relative risk for AI infection in the nomadic system was 1.08 times greater than the intensive system. Odds ratio of the study was 2.16; therefore, the incidence of infection with AI in the nomadic system was 2 times higher than in the intensive maintenance systems (95% CI: 0.33-14.31, p: 0.65).

## Discussion

Purbalingga district is one of the main centers of duck egg production in Central Java Province. It was selected as the study area as the district is quite representative of poultry market chains, including native backyard chickens, commercial farms, nomadic ducks, poultry collectors, and live birds markets. In this region, the majority of the maintenance systems are carried out by nomadic or free-grazing ducks. This system allows ducks to forage for themselves around the house, rice fields, rivers, and ponds; to cut production costs, farmers only offer a minimum of feed to these ducks [16,17].

Few farmers kept their ducks intensively, namely, by providing regular feed in a permanent cage setup, with or without a pool provided [16]. Under these circumstances, ducks are kept in cages for their whole lives and are fed twice a day using the farmer’s mixed feed.

The moving duck flock population monitored in this study was layer ducks, representing ducks that are generally kept for a longer production cycle compared to meat ducks. Exposure to LPAI H5 or HPAI virus is likely in layer ducks because of the extended production period [18]. Similar to a study by Tran and Yanagida [18], all moving duck flocks identified for this study were layer ducks, highlighting that layer ducks are the most common moving duck production system in the study area.

AI seropositive results taken from duck farms were likely exposed to field viruses. The majority of the ducks sampled were not vaccinated for AI, and only a few had been vaccinated against AI previously, or when the farmer bought the duck as day-old duckling or pullet (young duck), so they are considered as unvaccinated ducks. AI vaccination will provide an immune titer if done at least 2-3 weeks before the sample being collected. Seropositive results on HI but negative on RT-PCR can be caused by intermittent shedding [12]. A previous study reported that such AI viruses, for instance, LPAI H7, could be isolated from ducks [19]. Our study showed that HPAI H5 virus prevalence was 0% due to none of the samples detecting the H5 virus subtype positively. Other AI-HA virus subtypes may have been circulating in the duck population as we found from the RT-PCR results, no H5 positive results from matrix (MA) positive samples. Further study is necessary to investigate whether ducks play a role in non-H5 subtypes circulation in Indonesia.

The prevalence of influenza Type A virus (matrix gene positive) in adult ducks was around 10% and will continue to increase if no control program is instituted [17,20]. Henning *et al*. [21] stated that their study resulted in crude bird-level HPAI H5 antibody prevalence (HI titer ≥16) was about 15.5% in Vietnam and 5.3% in the nomadic duck population in Central Java, Indonesia. The previous study was conducted before the H5 Clade 2.3.2.1c increased in Indonesia; therefore, the antibody was moderately low. Infected ducks may exhibit no clinical signs, but they could excrete a high concentration of viruses that are pathogenic to other poultry species [21-23].

The study showed that the nomadic system poses a risk factor for AI virus transmission in the field. As stated from the previous studies [9,22,24], ducks play a key role in the maintenance of AI viruses because they act as a local reservoir and amplification host of viruses, which can be followed by secondary spread to other domestic poultry [22]. Most nomadic duck farms are classified as sector three because they have low biosecurity and are small to medium-sized [24]; therefore, it is challenging to control poultry diseases, especially AI, if they enter the flock. Farmers have experienced a decrease in egg production, up to 40%, due to infection with AI, resulting in the loss of their capital. The impact of H5N1 in the poultry sector has significantly affected duck farmers [25]; according to the farmers, the remunerations of raising ducks are only enough to cover the basic needs of their family.

The nomadic duck farming system is susceptible to disease when compared with the intensive rearing systems [16,17]. It also appears to be conducive for spreading and transmitting HPAI virus to other domestic birds because (1) flocks often move frequently and quickly over large distances, (2) flocks use many different scavenging areas, (3) flocks are exposed to numerous HPAI risk factors, such as having contact with other scavenging ducks, wild birds, and domestic animals, and (4) a large number of people are involved in transport and management of these flocks [12,22]. A previous study indicated that nomadic or scavenging ducks are a source of infection for other poultry and possibly, for humans [24]. Duck flocks graze in the same rice field where other potentially infected domestic or wild birds may have grazed; although not significantly different, this finding requires attention, especially when it concerns reducing the risk of disease infection in ducks, particularly in the nomadic system. When transporting nomadic flocks across sub-districts, district, or province borders may lead to the spread of AI viruses over relatively long-distances and due to a high density of animals kept and herded together; these result in increased stress levels which are likely to increase virus shedding and the risk of AI transmission [9,21,25,26].

The intensive rearing system can help prevent the transmission of HPAI because these farms have better biosecurity than the nomadic farming system, which might reduce virus transmission [23,26,27]. HPAI is still endemically circulating on Java Island, particularly in ducks and backyard chickens [23,28,29]; despite this endemic circulation, morbidity and mortality observed in outbreaks are still high, and neurological signs in ducks are frequently shown. The surviving duck population at Purbalingga District has seroconverted, indicating that virus replication occurred despite the absence of disease signs [9,30]. To anticipate the silent transmission and spread of such AI viruses, duck farmers with support from the government are encouraged to conduct vaccination and improved biosecurity practices [31,32].

## Conclusion

This study found that the seroprevalence of HPAI at the duck-level in Purbalingga was 54.69% and demonstrated that the nomadic duck farming system is more likely to acquire HPAI H5 infection than the intensive duck farming system. Other risk factors should be further investigated as the differentiation of the farming system is not significantly associated with HPAI H5 infection.

## Authors’ Contribution

WSN, TAK, and IY: Conceptualization. HW, GS, and IY: Data analysis and laboratory testing. WSN: Funding acquisition. IY: Data Collection. WSN, TAK, HW, GS, and IY: Resources. IY, HW, GS, WSN, and TAK: Writing, review and editing. All authors read and approve the final manuscript.
